# Conjugation with L,L-diphenylalanine Self-Assemblies Enhances *In Vitro* Antitumor Activity of Phthalocyanine Photosensitizer

**DOI:** 10.1038/s41598-017-13729-x

**Published:** 2017-10-13

**Authors:** Márcia I. Souza, Tatiana Prieto, Tiago Rodrigues, Fabio F. Ferreira, Francisco B. Nascimento, Anderson O. Ribeiro, Emerson R. Silva, Francesca Giuntini, Wendel A. Alves

**Affiliations:** 1 Centro de Ciências Naturais e Humanas, Universidade Federal do ABC, 09210-580 Santo André, SP Brazil; 2 Departamento de Biofísica, Universidade Federal de São Paulo, 04023-062 São Paulo, Brazil; 3 School of Pharmacy and Biomolecular Sciences, Liverpool John Moores University, Byrom Street, Liverpool, L3 3AF UK

## Abstract

We present the synthesis and characterization of new peptide conjugates obtained by hierarchical co-assembly of L,L-diphenylalanine (FF) and zinc phthalocyanine complexes (ZnPc) in water. Self-assembly capabilities under defined conditions were investigated by scanning electron microscopy, and photophysical properties were evaluated using UV-Vis and fluorescence spectroscopy. AFM observations demonstrated that these ZnPcs form different highly ordered arrays on the crystalline faces of the FF microplates and that surface roughness significantly changes with the presence of differently substituted phthalocyanine units. XRD assays showed that the overall molecular packing of the conjugates is organized according to a hexagonal symmetry, with ZnPcs hosted in the interstices of the peptide phase. *In vitro* photodynamic studies were conducted on human breast cancer MCF-7 cells to investigate both cellular uptake and cytotoxicity. It was shown that FF self-assemblies are not toxicity and enhance accumulation of ZnPc in MCF-7 cells, improving apoptotic cell death upon irradiation. Our findings demonstrate enhancement of ZnPc antitumor efficiency by FF conjugates and a proof-of-concept for new photosensitizer carriers based on peptide conjugates.

## Introduction

The ability of β-sheet-forming peptides to self-assemble into highly ordered nanostructures under specific conditions is well known and characterized, but great challenges still remain in the development of hierarchical self-assembly to achieve materials with designed properties^1^. It has been reported that materials based on L,L-diphenylalanine (FF) can self-assemble into various micro- or nanoarchitectures such as microtubes (MTs), nanotubes (NTs), nanowires (NWs), nanofibers (NFs), nanoribbons, ordered molecular chains and spherical vesicles^2^. Over the past few decades, several chemical and physical approaches have been developed for manipulating FF micro/nanoarchitectures by intervening during the process of supramolecular assembly.

The conjugation or association of suitable moieties to β-sheet-forming peptides can determine the occurrence of additional driving forces that have dramatic effect on the self-assembling process and on the structure of the resulting supramolecular architectures^3^. The structure and properties of dipeptide-based architectures can also be tuned by acting on their assembly pathways; many approaches to impart the desired characteristics to the self-assembled dipeptides have been explored, including the precipitation method, physical vapor deposition, vapor-transport, dielectrophoresis, solution-solid interface assembly, solution-solution interface assembly, enzyme-assistant self-assembly, and electrospinning processes^4–6^. These different strategies possess great potential for controlling the architectures and functionality of peptide-based materials.

According to recent reports, peptide-based hybrid nanostructures possess advantageous optical and photonic properties for novel applications in photonic/electronic devices^7^, light-harvesting materials and optical biosensing platforms^8^. We recently showed that the generation of reactive oxygen species (ROS) by the photosensitizer hypericin is enhanced upon complexation with FF micro/nanostructures (FF-MNSs)^9,10^, confirming that spatial arrangement plays a paramount role in determining photophysical behavior. In the same study, we demonstrated that the abundance of hydrophobic sites at the hypericin/peptide interface favors the creation of a nanoscopic environment in which non-polar species such hypericin, 1,3-diphenylisobenzofuran (DPBF) and molecular oxygen are brought together in a confined space. The supramolecular arrangement of FF behaves as a template for the spatial organization of the fluorophore, leading to lifetime increasing of molecules in the excited state. Our findings demonstrated that peptide-based nanostructures not only have a strong potential as photosensitizer carriers but they are also able to improve the photophysical behavior of the fluorophore.

To extend this concept to a new class of photosensitizers, we focused the current work on the ability of a series of ZnPc complexes to regulate and control the morphology of FF-based materials prepared by spontaneous co-assembly in water *via* a precipitation method. We investigated the distribution pattern of phthalocyanine onto the interfaces of FF-based micro/nanostructures (FF-MNSs) at the nanoscale and demonstrated that crystallization of hybrid FF-MNSs strictly follows a classical mechanism. According to our findings, new crystalline phthalocyanine layers are generated by 2D nucleation followed by growth through attachment of solute molecules, as shown by AFM and XRD observations. Interestingly, the association of ZnPc/FF-MNS conjugates does not affect singlet oxygen production of ZnPcs. Moreover, the fluorescence of ZnPc/FF-MNSs was highly stable under near-physiological conditions and in presence of the human breast cancer MCF-7 cell line. *In vitro* distribution study showed that FF-MNSs played an important role in the accumulation of ZnPc in tumor sites, providing an easy and efficient method to modulate the morphology and property of these functional molecules for enhanced photodynamic therapy.

## Results

### Preparation and morphology and conjugates

**Figure 1:**
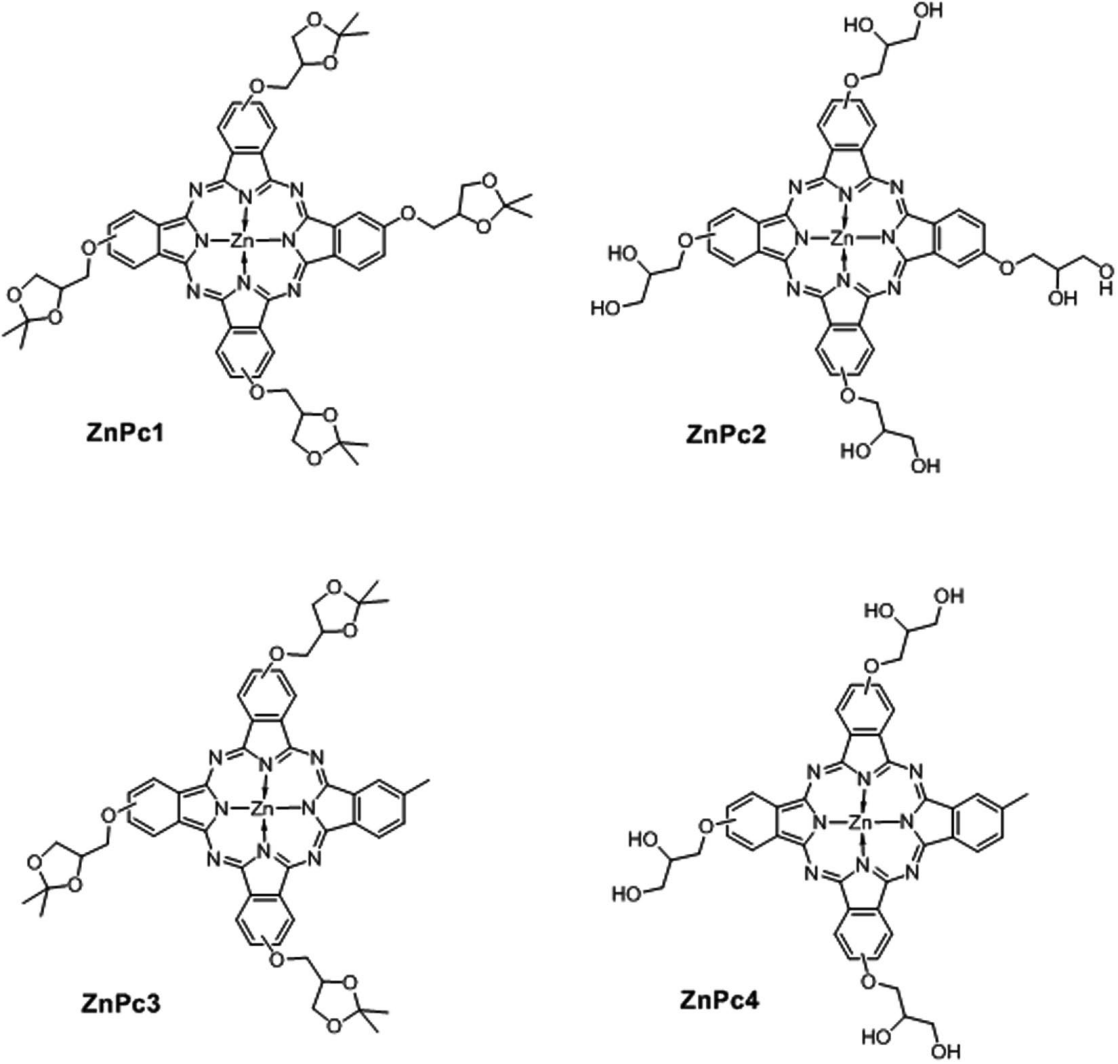
Zinc phthalocyanines used in this study.

The structures of phthalocyanines used in this work are shown in Fig. 1. Compounds ZnPc1 and ZnPc2 are tetra substituted with four identical groups (protected and non-protected glycerol, respectively), whereas compounds ZnPc3 and ZnPc4 are non-centrosymmetric (A3B type)^11^ and bear three glycerol substituents (protected and non-protected, respectively) and one methyl group.

**Figure 2:**
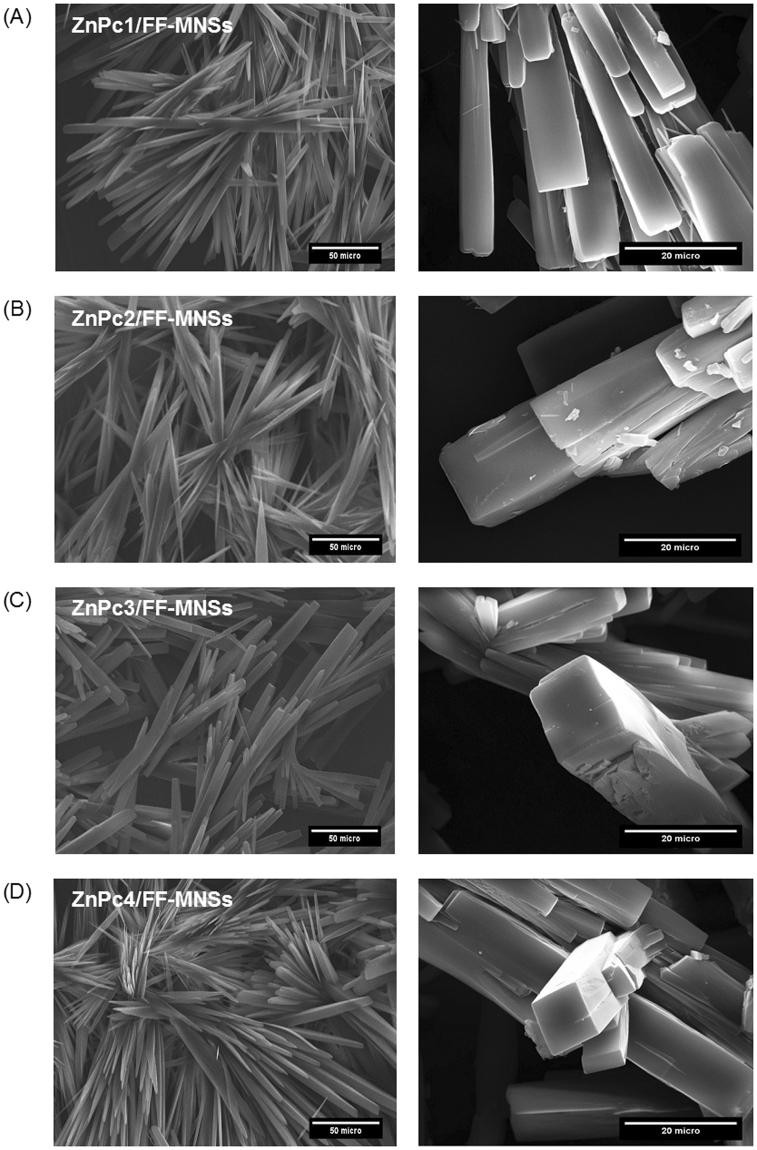
SEM images of FF-MNSs conjugated with different zinc(II) phthalocyanines: (**A**) ZnPc1, (**B**) ZnPc2, (**C**) ZnPc3, (**D**) ZnPc4.

The MNSs were prepared by precipitation using diluted solutions of FF and Zn(II) phthalocyanines in deionized water (see Materials and Methods for further details). This procedure led to the precipitation of nanostructures within a few minutes, and the morphology of the hybrids ZnPc/FF-MNSs was characterized by scanning electron microscopy (Fig. 2).

Addition of ZnPc induces remarkable morphological changes in the self-assembly in comparison to preparations where the dipeptide appears alone in solution. Indeed, under the same conditions (HFIP/methanol + water), the dimmer FF has been widely found to form tubular structures such as rods and tubes^1,2^ whereas the formation of rectangular microplates has been reported in HFIP/THF^12–14^. Herein, we have found that ZnPc/FF-MNSs hybrids self-assemble into highly anisotropic structures with a long axis and square cross-section endowed with sharp edges. It should be noted that this changeover on morphology has been found for all compounds investigated here, suggesting that the central aromatic macrocyclic ring strongly affects the self-assembly. A possible explanation for this could be related to additional π–π stacking interactions provided by large π-conjugated systems, which interplay with interactions mediated by peptide moieties (e.g., hydrogen bonds) into a synergic manner, thus altering the shape of the assemblies. Another noticeable feature common to all the ZnPc/FF-MNSs hybrids is the radial growth of the structures, which suggests the presence of nucleation centers from which the self-assembly occurs.

**Figure 3:**
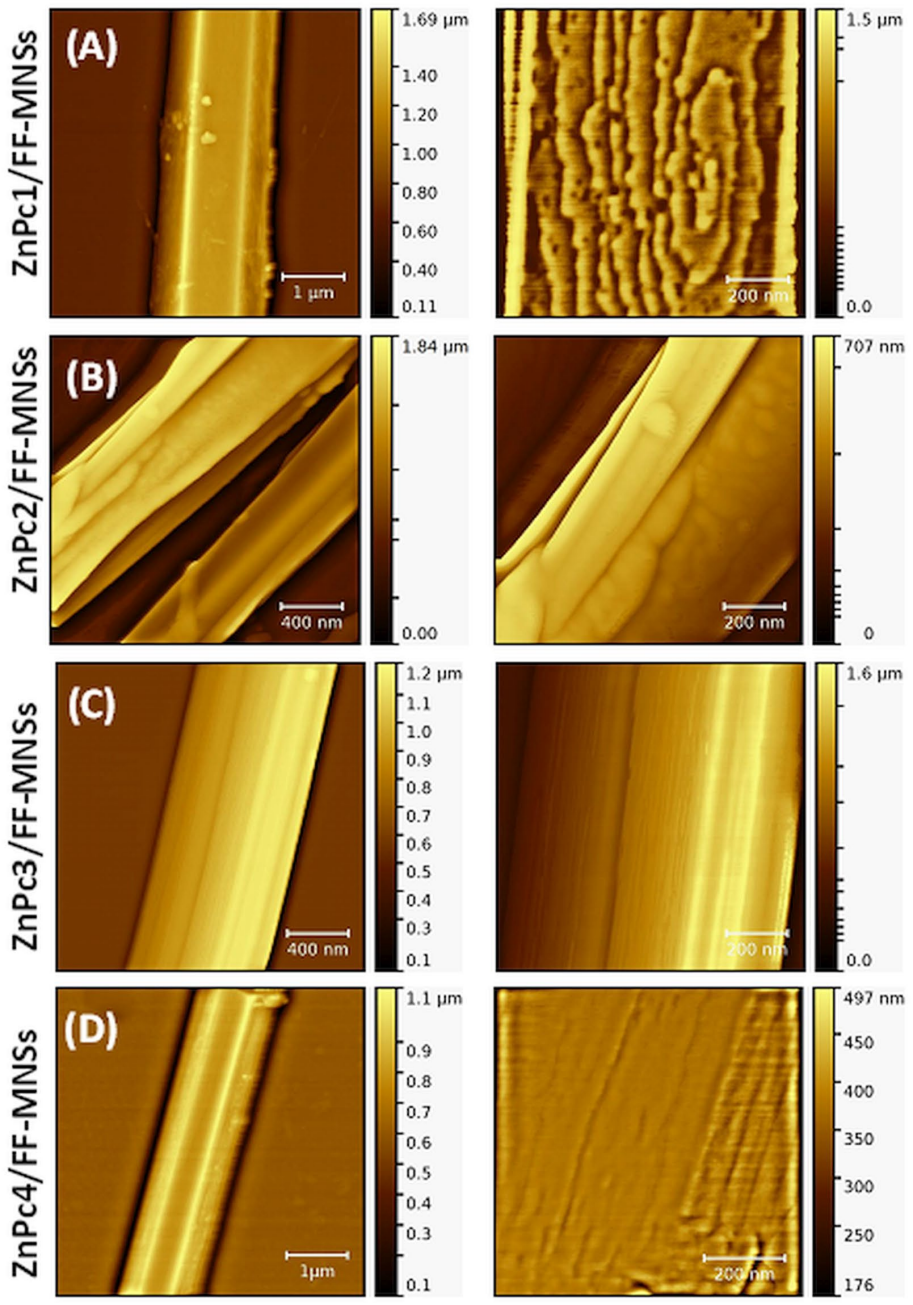
AFM topography images of ZnPc/FF-MNSs hybrids (**A**) ZnPc1, (**B**) ZnPc2, (**C**) ZnPc3, (**D**) ZnPc4. Left magnified image of the surface.

Surface characterization of ZnPc/FF-MNSs conjugates was performed by atomic force microscopy (AFM), as shown in Fig. 3. AFM images confirm the presence of sharp, well-defined edges on the assemblies, as previously attested by electron microscopy. However, at nanoscopic level, strong differences appear depending on the photosensitizer used to prepare the conjugate. The hybrid ZnPc1/FF-MNSs shows a superficial layer featured by remarkable terraces with sharp steps separating different plateaus, leading to surface roughness. The hybrid ZnPc2/FF-MNSs displays a surface characterized by granular structures and small clusters with a few tens of nanometers; however, even at high magnification, no surface pattern like the one observed for the hybrid ZnPc1/FF-MNSs could be identified for this conjugate. Hybrids containing ZnPc3 display longitudinal striations across their surfaces running parallel to the long axis of the self-assemblies. This surface behavior induces marked roughness at the interfaces, which likely implies higher surface areas for these conjugates. Structures formed with ZnPc4 show a surface characterized by large grooves with smooth cross-sectional profiles. No particular pattern is identified at the interfaces and at nanoscopic lengths the heights do not show large variations.

To provide quantitative description of surface roughness, AFM topography images were used for measuring the mean square roughness (Rq) coefficient. Measurements were made at twenty different regions of each sample and the corresponding average values are reported in Table 1 together with the corresponding standard deviation of the mean. The highest roughness coefficient was found for the hybrid prepared with the noncentrosymmetric ZnPc3 compound bearing protected glycerol units.

**Table 1:** Surface roughness values for ZnPc/FF-MNSs.

Sample	Rq
ZnPc1/FF-MNSs	10.1 ± 0.5 nm
ZnPc2/FF-MNSs	11.7 ± 0.9 nm
ZnPc3/FF-MNSs	15.7 ± 1.2 nm
ZnPc4/FF-MNSs	11.3 ± 1.1 nm

**Figure 4:**
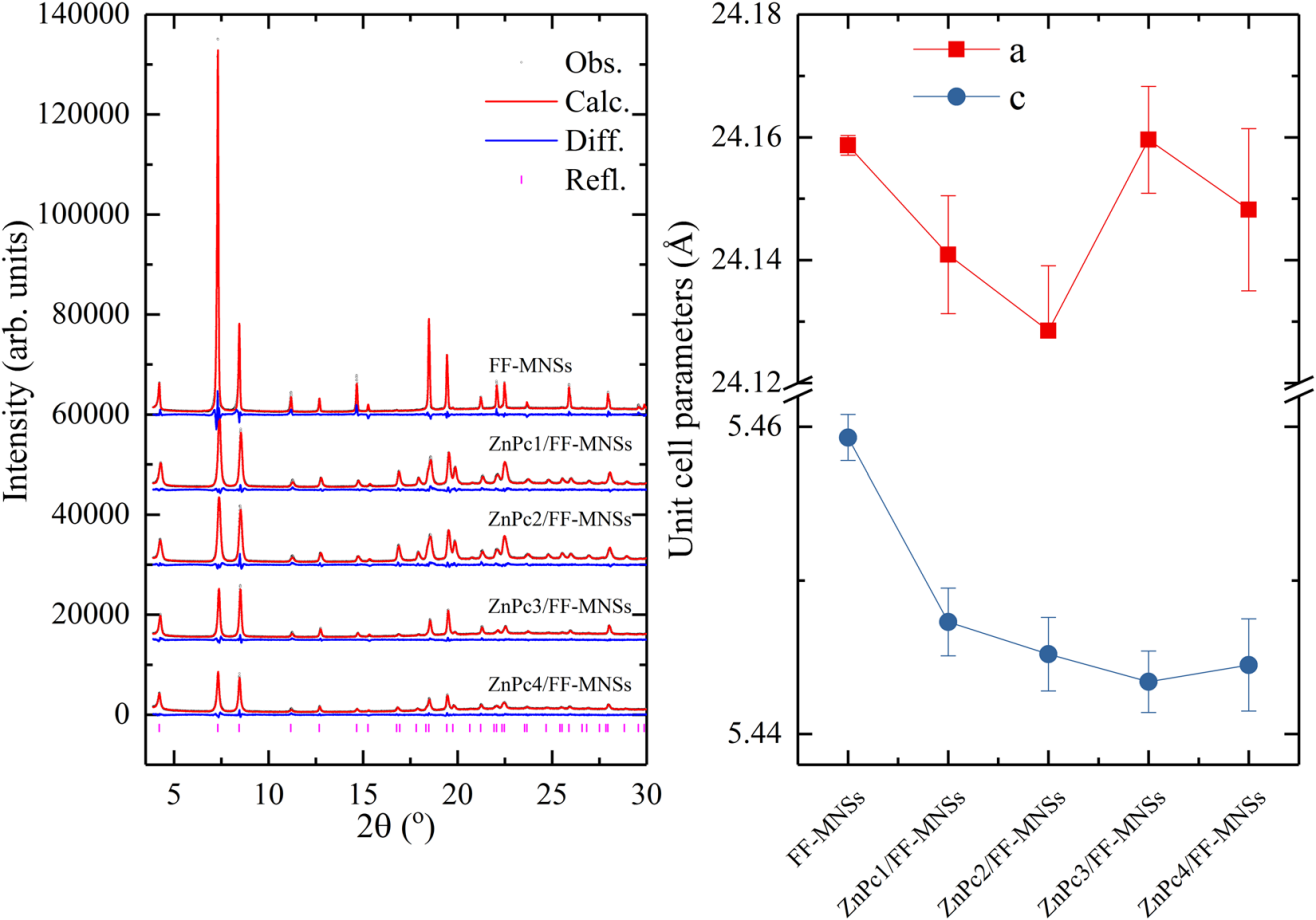
(left) Rietveld plots from the final refinements. Black open circles represent the observed patterns; red lines indicate the calculated patterns; blue lines at the bottom of each diffractogram display the difference between observed and calculated patterns. The magenta vertical bars represent the Bragg reflections of the FF crystal structure. From top to bottom: FF-MNSs, ZnPc1/FF-MNSs, ZnPc2/FF-MNSs, ZnPc3/FF-MNSs and ZnPc4/FF-MNSs. (right) Variation of unit cell parameters from FF-MNSs to ZnPc4/FF-MNSs.

Investigation of the X-ray diffraction (XRD) patterns of the ZnPc/FF-MNSs hybrids showed that all of them adopt a hexagonal crystal structure (*P*6_1_)^15^, although the samples all displayed different fine morphologies (Fig. 4). We did not observe minor or secondary phases, attesting that polymorphs observed in microscopy assays above are in fact hybrid conjugates formed between FF and the photosensitizers. The major differences observed in the experimental diffraction patterns (magenta line in Fig. 4) are mainly due to the effect of preferred orientation of the crystallites and to the disorder of the water molecules within the FF channels (see Figure S2). This inference was confirmed by Rietveld refinements, as shown in Fig. 4 ^16,17^, in which we kept the FF atoms within the unit cell fixed and allowed only the water molecules and unit cell parameters to move freely. The agreement between the calculated and observed patterns was improved using this strategy. We observed that different ZnPcs did not alter the distance between the crystallographic planes of hybrid materials. The major difference is observed in the cell parameter *c* and unit cell volumes, when compared to the FF-MNSs in the absence of ZnPcs, which indicates that the presence of phthalocyanine moieties decreases the distance between the crystallographic planes of the FF-MNSs. It is expected that phthalocyanine moieties can be found between the hexagonal planes indexed as belonging to the *P*6_1_ space group. This result corroborates previous work of our research group that showed intercalation of guest photosensitizers into FF structures takes place along the longitudinal axis of the structures^9^.

#### Photophysical characterization

**Figure 5:**
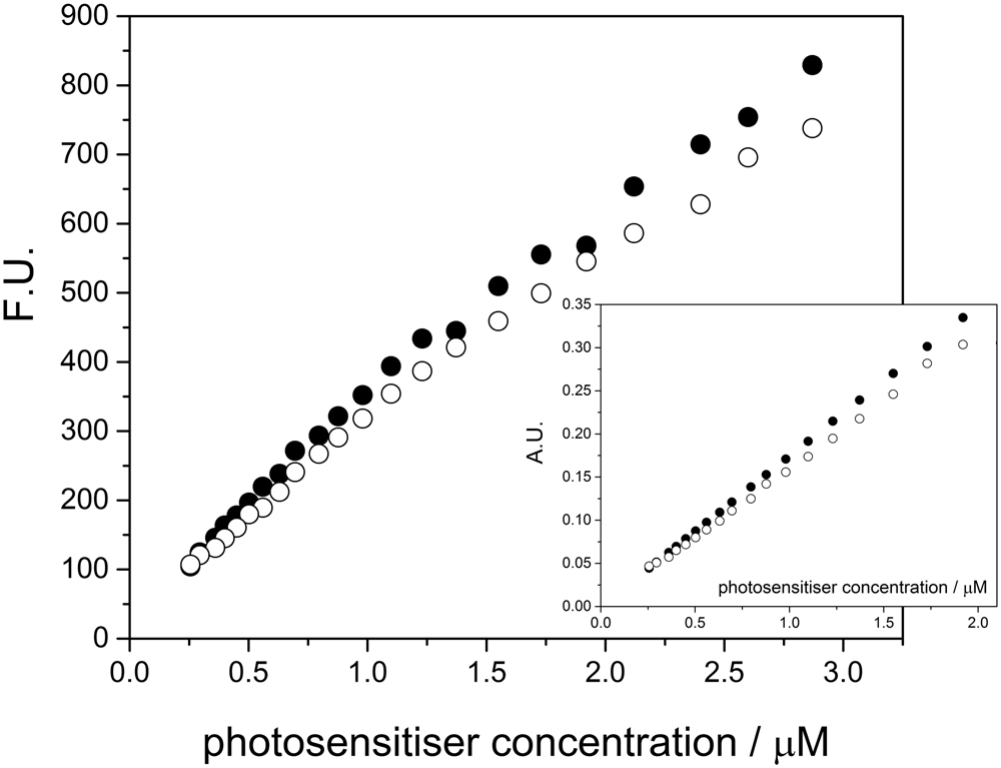
Representative variation of the emission and absorption (inset) maxima of ZnPc3 (close circles) and ZnPc3/FF-MNSs (open circles) with chromophore concentration. Emission was measured at 693 nm (λ_exc._= 615 nm) and absorption at 680 nm.

**Figure 6:**
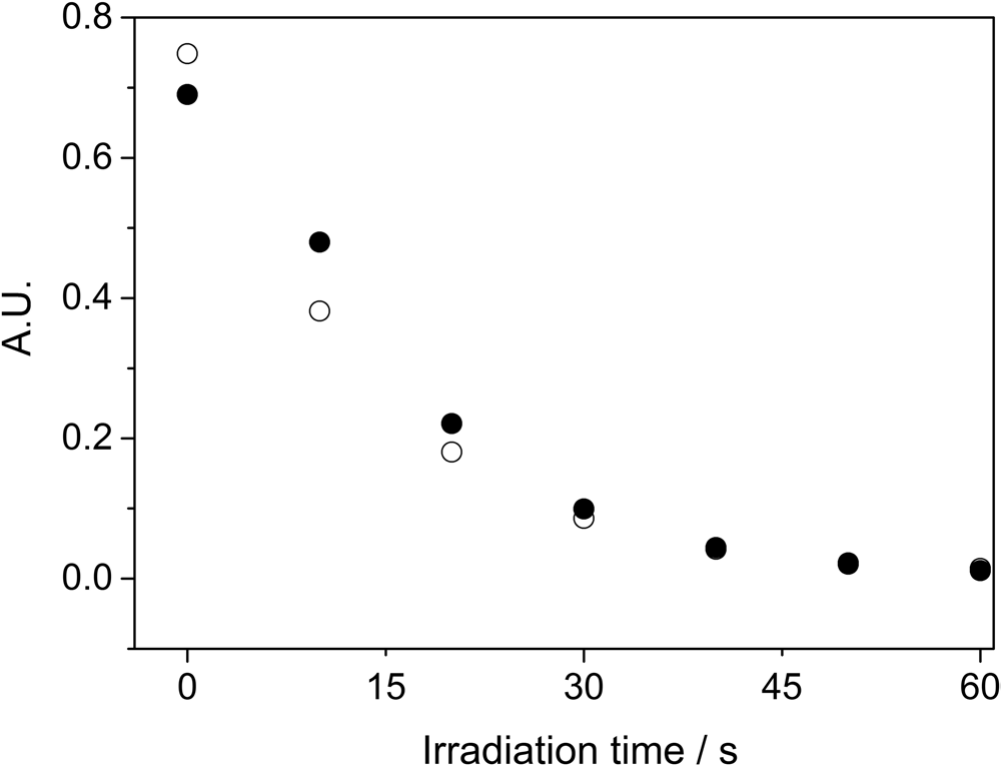
Representative decay rates of absorption intensity of DPBF (417 nm) following photosensitization by 0.12 mM ZnPc3 (close circles) and ZnPc3/FF-MNSs (open circles).

The absorption and fluorescence spectra of ZnPcs and ZnPc/FF-MNSs are reported in Figure S3. Inclusion of the phthalocyanine in the MNSs does not cause a shift in the absorption, but the electronic emission spectra displayed a bathochromic shifts as a result of different morphology that may induce minor interactions between the chromophores. Both absorption and emission increase linearly with concentration within the range studied, either for ZnPcs or ZnPc/FF-MNSs (Fig. 5 for ZnPc3 and corresponding MNSs; remaining graphs are provided in the SI). ZnPcs chromophore in the excited triplet state transfers energy to molecular oxygen with high efficiency, generating singlet oxygen and other reactive oxygen species (ROS)^18^. Evaluation of the singlet oxygen generation efficiency of the different ZnPc/FF-MNSs hybrids was assessed by following the bleaching of the molecular trap 1,3-diphenylisobenzofuran (DPBF) upon reaction with singlet oxygen^19^. Our findings indicate that the association with FF-MNSs does not affect singlet oxygen production of ZnPcs. Figure 6 shows a representative example of the DPBF absorbance intensity decay *versus* irradiation time.

#### *In vitro* photodynamic activities against breast cancer cells

Since conjugates made with the ZnPc3 phthalocyanine exhibited the highest surface roughness at nanoscale, we have chosen this hybrid for testing both cytotoxicity and photodynamic activities against breast cancer cells. This morphological characteristic leads to higher interface area in these materials; thus, these conjugates are presumably more efficient to provide interactions with cell membranes^20^. In addition, the ZnPc3 compound has a high partition coefficient (log P) than the other phthalocyanines, as shown in Table from the literature^11^. In this case, we have observed that a number of protected glycerol groups improved the hydrophobicity of the compounds, which can be associated with higher tendencies to cross the membrane lipid bilayers resulting in increased uptake of the compounds into cells, as shown *in vitro* photodynamic activities against breast cancer cells by using these compounds^11^. However, the ZnPc3 alone showed no interaction with MCF-7 cells when compared to the ZnPc1 compound, which had an efficiency of 80%. The reason is that the extremely lipophilic phthalocyanines tend to form aggregates in media a thus prevent plasma membrane diffusion. Thus, the choice of the ZnPc3/FF-MNSs hybrid system as a photosensitizer carrier could evidence the application of peptide nanostructures for effective delivery of photosensitizers into cells.

First, a concentration-response curve was performed to screen the cytotoxicity of the ZnPc3/FF-MNSs system in the human breast cancer MCF-7 cells. Cell viability was determined after 10 minutes of irradiation and 24 hours of incubation in the dark, using the neutral red uptake assay. For comparative purposes, cell viability was also determined in the dark, i.e., using the same experimental conditions except for the irradiation step^21,22^.

Also, ZnPc3/FF-MNSs concentrations were selected in order to maintain the same molar concentration of ZnPc3 as when it was used alone. The ZnPc3/FF-MNSs system decreased the MCF-7 cell viability in a concentration dependent manner and this effect was significantly exacerbated under irradiation, reaching 50% of cytotoxicity at 0.2 mg/mL *versus* only 20% in the dark (Supplementary material, Figure S5).

**Figure 7:**
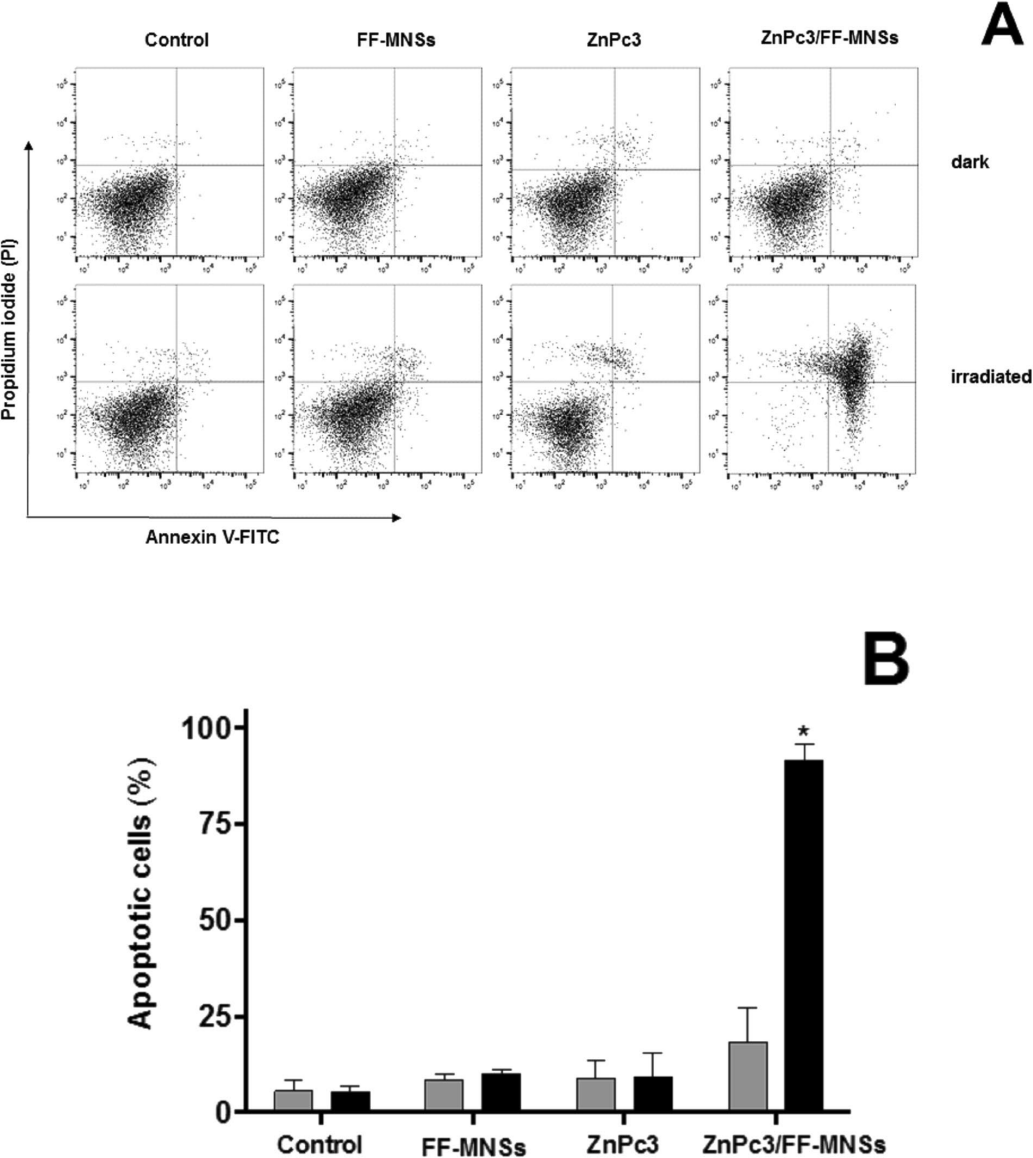
Cell death profile investigated by annexin V-FITC and PI double staining flow cytometric analyses. MCF-7 cells (6 × 10^5^ cells/experiment) were incubated with 0.42 µmol/L ZnPc3, 0.2 mg/mL FF-MNSs and 0.2 mg/mL ZnPc3/FF-MNSs for 2 hours and irradiated at 660 nm for 10 minutes. Flow cytometry was done 24 h after irradiation and compared to the same experimental conditions in the dark. (**A**) Representative dot plot panels of one experiment (An^−^/PI^−^ viable cells An^−^/PI^+^ suggests necrosis; An^+^/PI^−^, early apoptosis; An^+^/PI^+^, late apoptosis or apoptosis followed by necrosis), and (**B**) quantification of the percentage of cell population in each quadrant obtained from two independent experiments done in duplicate. *One-way ANOVA followed by Tukey’s post hoc test showing statistically different from control (p < 0.05).

The type of cell death, i.e. apoptosis or necrosis, induced by ZnPc3/FF-MNSs has been investigated by using the annexin V-FITC and propidium iodide (PI) double-staining flow cytometry, and the representative dot plots are presented in panel A and the respective quantification of the replicates in panel B. Annexin V-FITC positive staining indicates the phosphatidylserine externalization to the outer leaf of plasma membrane that occurs in apoptosis, and this can be experimentally observed with dots in the right (up and bottom) quadrants. Propidium iodide (PI) positive staining refers to plasma membrane rupture during the necrotic processes and it is indicated by plots in the up (left and right) quadrants^23^. As observed in the Fig. 7A, a double positive population was achieved only for ZnPc3/FF-MNSs upon irradiation, which indicates simultaneous staining with annexin V-FITC and PI after 24 h incubation with the cells, indicating late apoptosis or apoptosis followed by secondary necrosis. Such staining pattern is often observed with drug-induced cell death at prolonged incubation times, since the externalization of phosphatidylserine is an early event in the apoptotic cascade that can be followed by disruption of plasma membrane due to persistent chemical stimulation. The quantification of the annexin V-positive cells, considering all replicates, is presented in Fig. 7B confirming that irradiated ZnPc3/FF-MNSs affected significantly cell viability and induced apoptosis in MCF7 breast tumor cells.

**Figure 8:**
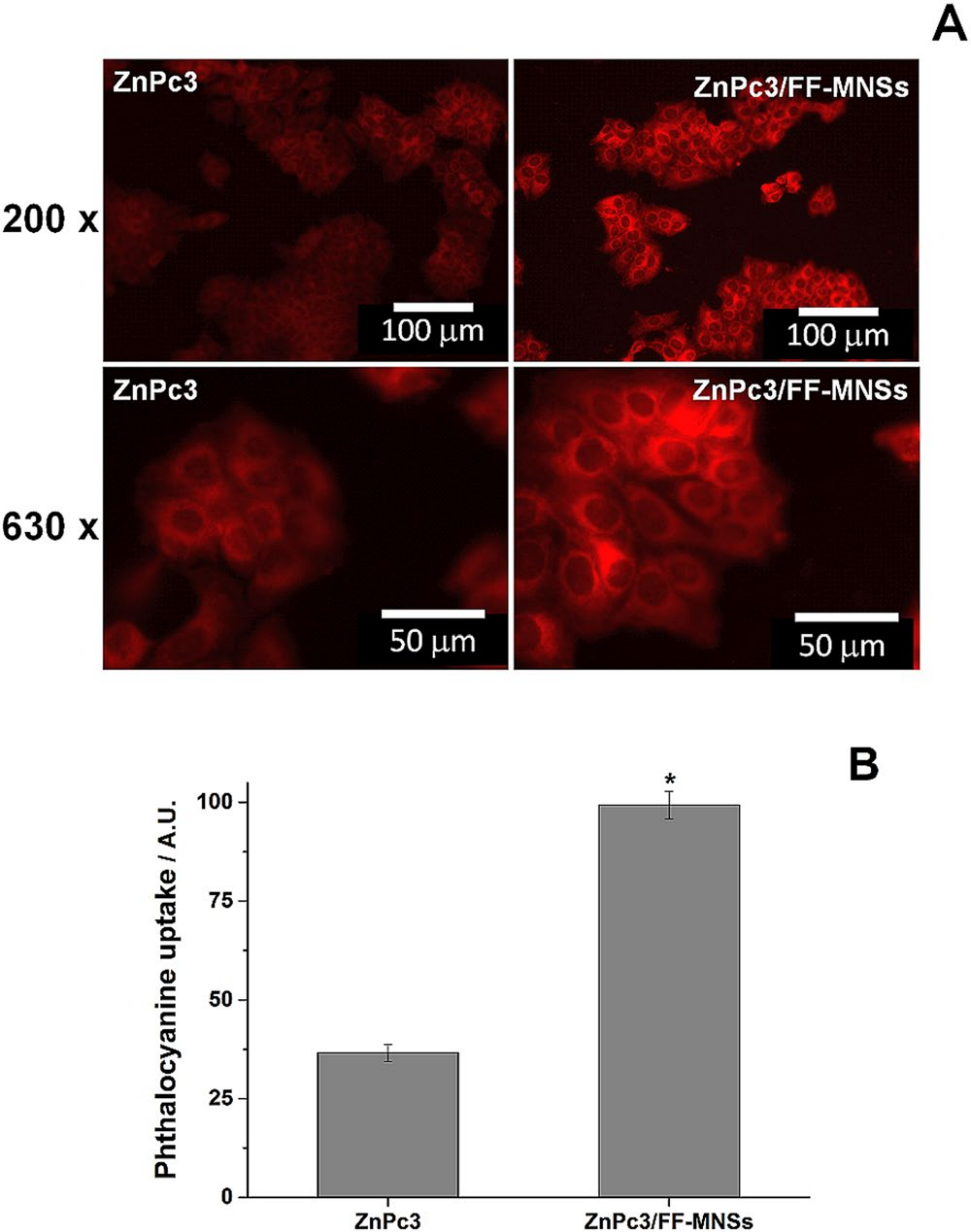
ZnPc3 uptake by MCF-7 cells. (**A**) Representative image of fluorescence emission in MCF-7 cells after 2 h incubation with 0.62 µmol/L ZnPc3 (left panels) and 0.3 mg/mL ZnPc3/FF-MNSs (right panels). Upper and bottom panels refer to images obtained under 200 and 630x magnification, respectively, in a Leica DMI 6000B microscopy system using Y5 filter with excitation at 675 nm. Scale bars: 50 µm (200x) and 20 µm (630x). (**B**) Fluorescence quantification was performed at 630x magnification using Leica Application Suite software (LAS, v. 3, Leica Microsystems, Germany). *One-way ANOVA followed by Tukey’s post hoc test showing statistically different from control (p < 0.05).

The high fluorescence emission yield of free zinc phthalocyanine molecules or associated with FF-MNS was used to estimate the ZnPc3 cellular uptake. Cells were grown in optical glass bottom dishes followed by incubation with the nanostructured systems. After washing, fluorescence emission was acquired in the same experimental conditions for all samples at 200 and 630x magnifications. The images presented in the Fig. 8A demonstrated higher fluorescence emission in cells incubated with ZnPc3/FF-MNSs than in those incubated with ZnPc3, indicating that MNS system promoted a higher uptake of phthalocyanine. Also, the absence of a punctuated pattern of fluorescence suggests that only the uptake of the dye and not the entire nanostructured system. The fluorescence emission quantification was performed only in the imagens obtained at 630x  magnification and presented in the Fig. 8B. As observed, the fluorescence intensity of the cells treated with ZnPc3/FF-MNSs was 60% higher than the fluorescence intensity of cells treated with free ZnPc3. This can explain, at least partially, the higher cytotoxicity observed by irradiated ZnPc/FF-MNSs.

From the findings presented above, one observes that FF-MNSs do not increase the generation of ROS directly or alter the spectroscopic behavior of ZnPc. However, the clear enhancement of cell death together to improvement of fluorescence in the vicinities of cell membranes demonstrated in our biological assays, are evidence for the capabilities of these conjugates to assist access of the photosensitizer to the cells and promote photoinduced death. In this manner, FF-MNSs behaved as efficient delivery agents for ZnPc photosensitizers, whereas simultaneously showing remarkably low toxicity^24^. The ability of the micro/nanostructure system to enhance the accumulation of the ZnPc sensitizer in the cells is attributed to their ability to establish van der Waals interactions between the biomacromolecule corona and the cell membrane^25^. In our work, the high ZnPc3 fluorescence shown by cells treated with ZnPc3/FF-MNSs and the indication that necrosis is the predominant pathway of cell death following ZnPc/FF-MNSs -PDT support this hypothesis.

## Discussion

In summary, a method for efficient incorporation of a series of tetrasubstituted ZnPcs in FF nanotubes was presented. The presence of the phthalocyanines affected the morphology of the nanostructures obtained from the FF nanotubes, leading to sharp-edged shapes. The pattern and nature of the substituent was shown to affect the surface roughness and the XRD fingerprint of the micro/nanorods. Additionally, the incorporation of Zn phthalocyanine chromophores into FF-MNSs does not directly enhance the photodynamic efficiency of the photosensitizer (i.e., neither singlet oxygen production efficiency increase nor does the aggregation behavior vary). In despite this observation, FF-based conjugates performed better in the photodynamic inactivation of MCF-7 cells compared to the free phthalocyanine, by triggering apoptosis followed by secondary necrosis. Fluorescence images indicate higher uptake of the photosensitizer, and flow cytometry shows evidence of membrane damage, suggesting that the predominant pathway of cell death is necrosis. Studies to further understand the enhanced photoinactivation efficiency of peptide structure-supported phthalocyanines are currently underway and will explore the role that the waveguide behavior of these 1D assemblies play in this process.

## Materials and Methods

### Reagents

L,L-diphenylalanine and 1,1,1,3,3,3-hexafluoro-2-propanol (HFP) were purchased from Sigma-Aldrich (USA). 1,3-diphenylisobenzofuran, dimethyl sulfoxide (DMSO) and methyl alcohol were purchased from Synth (Brazil). Zinc phthalocyanine was synthesized as reported elsewhere^11^. Ultrapure water (R > 18 mol L^−1^ Ωcm^−1^) was obtained from a Milli-Q system. All reagents were analytical grade and used without further processing.

### Preparation of ZnPc/FF-MNSs

Eleven samples containing 3 mg of FF in 30 μL HFP were prepared into Eppendorf tubes. To the resulting solutions, different aliquots of a 1.3 × 10^−3^ M solution of ZnPc in DMSO were added to obtain final concentrations of fluorophore ranging from 2.4 to 8.3 µM. The samples were mixed using a Vortex mixer for several minutes, 150 μL of ultrapure water were added and the solutions were mixed again with a Vortex stirrer. After 20 minutes, precipitation of the hybrid ZnPc/FF-MNSs was observed. The samples were kept in the dark, at room temperature, left to dry in air over the course of a few days and then stored at 7 °C until used. It was assumed that the total amount of the peptide available was converted into FF-MNSs and that all ZnPc was incorporated in the structures^10^.

### X-ray diffraction (XRD)

XRD patterns of powdered samples were recorded at room temperature on a D8 Focus Discover diffractometer (Bragg−Brentano configuration). The beam was provided by a Cu-target source, λ  = 0.154 nm, operating at 40 kV/30 mA. The 2θ range was scanned between 5 and 30°. For XRD measurements, we have measured a silicon powdered sample provided by NIST (SRM 640d) and the zero point of the diffractometer was calibrated by using a peak-calibration routine implemented in the software manufactures.

### Microscopy

Scanning electron microscopy (SEM) images were obtained using a JEOL FEG-SEM JSM 6330 F or a JEOL LV-SEM microscope at the LNNano (Brazilian Nanotechnology National Laboratory, Campinas).

### Atomic force microscopy

AFM imaging was carried out on a Digital Instruments Nanoscope III at the LNNano (Brazilian Nanotechnology National Laboratory, Campinas), operating in tapping mode with tip frequencies around 250 kHz. All images were recorded with 512 × 512 pixels. Samples were prepared by casting droplets from solutions containing ZNPcs/FF-MNSs onto cleaned glass microscopy slides and letting them dry for a couple of hours under nitrogen stream. The samples were first inspected with an optical microscope attached to the AFM and, once isolated peptide microstructures were identified, probe scanning was performed in areas with 5 × 5 μm². Adhesion of ZnPcs to FF-MNSs was investigated in detail by probing zones with 1 × 1 μm² directly onto the surface of peptide microtubes. Image treatment and visualization were performed using the software Gwyddion and surface roughness were estimated by determining root mean squared coefficients (*R*_*q*_) from twenty random height profiles.

### Photophysical assays

The photooxidation of DPBF was monitored by recording its UV/vis absorption spectra on a VARIAN 50 scan spectrophotometer. An illumination diffusion table equipped with a yellow LED (emission band centered on 640 nm) was used as the light source. An LED beam (640 nm) was coupled to the system for irradiation. Solutions of ZnPcs or ZnPc/FF-MNSs in DMSO (2.4–8.3 µmol) were irradiated for 10 minutes at 10 mW/cm^2^ (reaching a fluence of 6 J/cm^2^), and absorbance spectra over the range 350–700 nm were recorded every 10 s. The intensity decrease of the band at 417 nm was used as an indication of DPBF photo bleaching^26^.

### Cell culture

The human breast cancer MCF-7 cell line (ER+) was purchased from Rio de Janeiro Cell Bank (BCRJ 0162) and cultured in RPMI 1640 medium (Sigma-Aldrich, MO, USA), supplemented with 10% (v/v) heat inactivated fetal bovine serum (FBS) (GIBCO-Invitrogen Corp., Grand Island, NY, USA), 100 U/mL penicillin (GIBCO-Invitrogen Corp.) and 100 µg/mL streptomycin (GIBCO-Invitrogen Corp.). Cells were maintained in a humidified atmosphere with 5% CO_2_ at 37 °C (Sanyo MCO-20AIC incubator; Sanyo Electric Co., Ltd., Osaka, Japan). Cells were sub-cultured every 72 h growth after 0.25% trypsin-EDTA solution treatment. Cell viability was assessed before the beginning of each experiment by the trypan blue-dye exclusion method, and viable cells were cultivated at 24 h to achieve exponential growth.

### Cells photosensitization

Cells were incubated with ZnPc3 or ZnPc3/FF-MNSs for 2 h in the presence of 2% FBS to prevent aggregation and washed twice in PBS solution prior to irradiation to ensure that only the internalized photosensitizer was excited. The irradiation process were done in a PBS solution where cells were irradiated for 10 minutes using a Biotable^®^ light source (developed by the Technical Support Laboratory, São Carlos Institute of Physics, University of São Paulo) equipped with an array of 24 LEDs (Edson Opto Corporation, Taiwan) emitting at 660 nm, providing a uniform distribution of light with a fluency rate of 36.5 mW/cm^2^ to the whole plate^27^. Following irradiation, PBS was exchanged with RPMI medium supplemented with 10% FBS and the cells were returned to the normal cellular growth conditions. Assays were performed 24 h after irradiation as detailed below.

### Cell viability assay

The effect of FF-MNSs/ZnPc on MCF-7 cell viability was screened through the neutral red uptake assay. MCF-7 cells (4 × 10^5^ cells/cm^2^) were seeded in a 96-well microplate (0.2 mL final volume) for 24 h and then exposed to increasing concentrations of ZnPc3/FF-MNSs (or FF-MNSs, as control). For the photosensitization, cells were irradiated for 10 minutes (as detailed above) and then incubated in a humidified atmosphere with 5% CO_2_ at 37 °C in the dark for 24 h. After washing twice with PBS, 0.1 mL of 50 mg/mL neutral red (3-amino-7-dimethylamino-2-methylphenazine hydrochloride, Sigma Chemical Co.) solution was added to each well followed by incubation for 3 h. After washing twice again with PBS, 0.1 mL of fixing solution (3% formaldehyde, 1% CaCl_2_) and 0.1 mL of extracting solution (1% glacial acetic acid in ethanol) were added, and the cells were kept under gentle agitation in a plate shaker for 10 minutes at room temperature. After that, the absorbance at 540 nm was recorded in a microplate reader (Synergy HT, BioTek Instruments, Winooski, VT, USA), and the percentage of viable cells (mean of three independent experiments) was calculated in relation to control, considered as 100%. Dark experiment corresponds to cells under the same treatment except by the irradiation step.

### Apoptopsis/necrosis detection

In order to investigate the type of cell death elicited by ZnPc3/FF-MNSs, an annexin V-FITC/iodide propidium double staining flow cytometric analysis were performed. Cells (3 × 10^5^/cells/cm^2^) were grown for 24 h in 24-well microplates. The incubation and irradiation steps occurred in the presence of 0.42 µM of ZnPc3, 0.2 mg/mL ZnPc3/FF-MNSs an.d 0.2 mg/mL of FF-MNSs. Cell suspensions were irradiated for 10 min and then incubated for 24 h (as detailed above), after which cells were harvested using trypsin/EDTA solution, centrifuged (160 × *g* for 10 min), and suspended in 50 µL of the binding buffer (0.14 M NaCl, 2.5 mM CaCl_2_, 0.01 M HEPES, pH 7.4). Then, 3 μL annexin V-FITC (BD Biosciences, San Jose, CA, USA) and 3 μL PI (BD Biosciences) were added to the cell suspension and the mixture was incubated in the dark at room temperature for 20 min. After the addition of 0.3 mL of binding buffer to each tube, the fluorescence measurements were performed in a FACS Canto II flow cytometer (BD Biosciences), acquiring 10,000 events per sample. Data analysis and graphs were done using Flow Jo vX.0.7 software (Ashland, OR, USA).

### Cellular uptake of photosensitizer

MCF-7 cells (1 × 10^4^ cells/cm^2^) were grown in supplemented RPMI medium in glass bottom dishes (35-mm dishes with a 0.17-mm thick cover glass on the bottom; Greiner Bio-One, Germany) for 24 h. After washing, cells were incubated in RPMI medium supplemented with 2% FBS with 0.62 µM ZnPc3, 0.3 mg/mL ZnPc3/FF-MNSs or 0.3 mg/mL FF-MNSs for 2 h. Cells were washed with calcium- and magnesium-free saline solution, and images were acquired on Leica AF6000 microscopy system (Leica Microsystems, Germany) using a fluorescence buffer (1.5 mM CaCl_2_, 130 mM NaCl, 5.6 mM KCl, 0.8 mM MgSO_4_, 1 mM Na_2_HPO_4_, 25 mM glucose, 2 mM HEPES and 2.5 mM NaHCO_3_–pH 7.3). Fluorescence emission was recorded using the filter cube Y5 (Leica Microsystems), with 620 nm excitation and 60 nm bandpass, 660 nm dichroic mirror and 700 nm emission with 75 nm bandpass. Differential interference contrast microscopy images were acquired by using an HCPL Apo 20× /0.7 and HCX PL Apo CS. 63x/1.40-0.60 OIL numerical aperture plan apochromatic objective coupled to an ultrafast Leica DFC365 FX digital camera (Leica Microsystems, Germany). The fluorescence intensity, relative to the amount of photosensitizer cell uptake, was quantified using the Leica Application Suite program (LAS, v. 3, Leica Microsystems).

## Electronic supplementary material

Supplementary Information
