# Supplementary material for: Conjugation with L,L-diphenylalanine Self-Assemblies Enhances In Vitro Antitumor Activity of Phthalocyanine Photosensitizer
Source: Sci Rep. 2017 Oct 13;7:13166. doi: 10.1038/s41598-017-13729-x (PMC5640658; doi:10.1038/s41598-017-13729-x)
Supplement: Supplementary file 1 — Supplementary Information [file 41598_2017_13729_MOESM1_ESM.doc]

**ELECTRONIC SUPPLEMENTARY INFORMATION**

**Conjugation with L,L-diphenylalanine Self-Assemblies Enhances *In Vitro* Antitumor Activity of Phthalocyanine Photosensitizer**

**Márcia I. Souza,**^1^ **Tatiana Prieto,**^1^ **Tiago Rodrigues,**^1^ **Fabio F. Ferreira,**^1^ **Francisco B. Nascimento,**^1^ **Anderson O. Ribeiro,**^1^ **Emerson R. Silva,**^2^ **Francesca Giuntini,**^3^ **Wendel A. Alves**^1^*

^1^Centro de Ciências Naturais e Humanas, Universidade Federal do ABC, 09210-580, Santo André, SP, Brazil.

^2^Departamento de Biofísica, Universidade Federal de São Paulo, 04023-062, São Paulo, Brazil.

^3^School of Pharmacy and Biomolecular Sciences, Liverpool John Moores University, Byrom Street, Liverpool L3 3AF, UK.

*Corresponding author: [wendel.alves@ufabc.edu.br](mailto:wendel.alves@ufabc.edu.br)


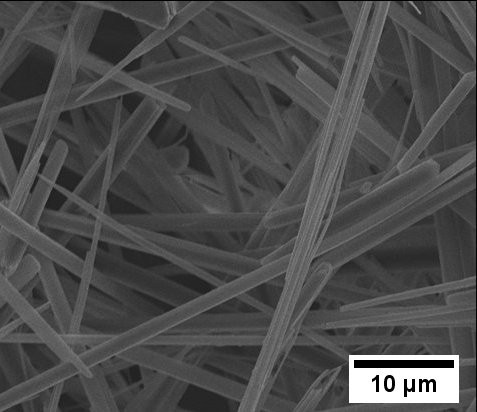


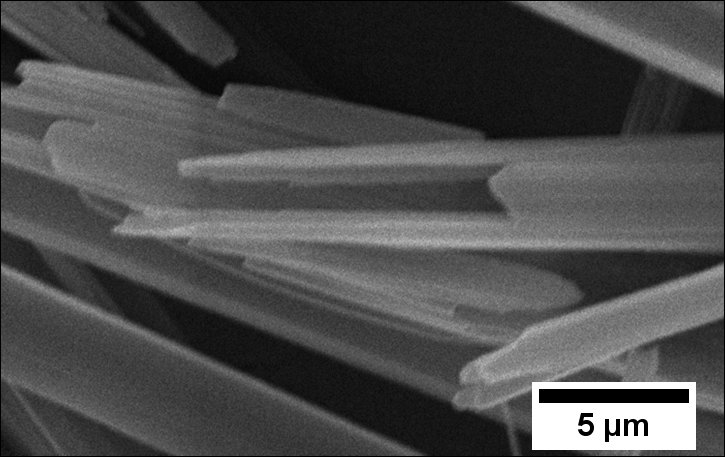


**Figure S1.** SEM images of diphenylalanine nanostructures in the absence of phthalocyanines using a mixture of HFIP and aqueous solution.


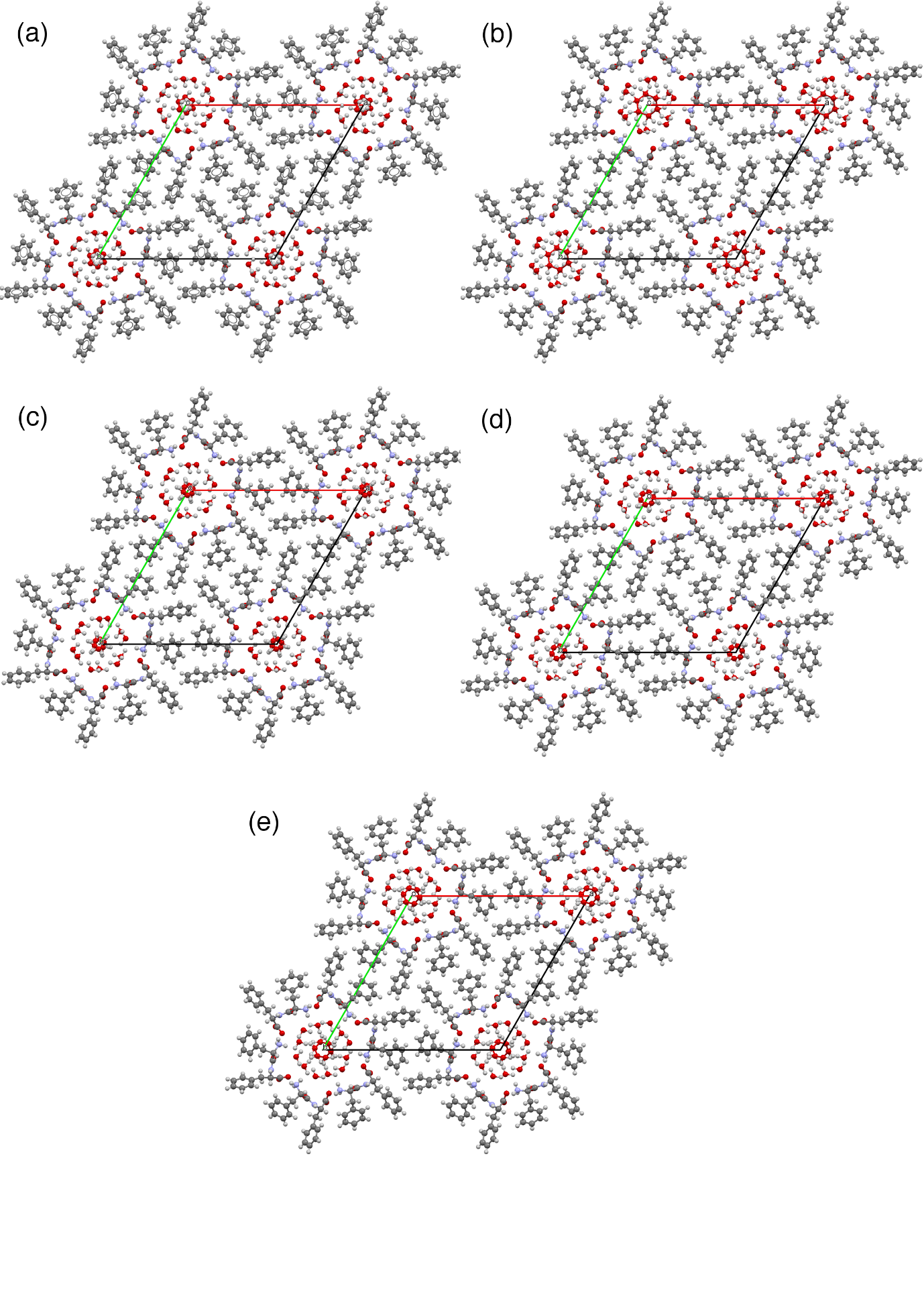


**Figure S2.** Packing of the crystal structures of (a) FF-MNSs, (b) ZnPc1/FF-MNSs, (c) ZnPc2/FF-MNSs, (d) ZnPc3/FF-MNSs and (e) ZnPc4/FF-MNSs displaying the slight variation of the water molecules arrangement along the c-axis.

**Absorption spectra**

**Fluorescence spectra**

**Figure S3.** The absorption and fluorescence spectra of ZnPcs and ZnPc/FF-MNSs.

**Figure S4.** Effect of concentration on the absorption and emission maxima.

**Figure S5. Effects of FF-MNSs and ZnPc3/MNSs on MCF-7 cell viability.** Cells (4 × 10^5^ cells / well) were incubated for 2 h with increasing concentrations of FF-MNSs (A) or ZnPc3/FF-MNSs (B) in the dark (black square) or irradiated at 660 nm for 10 minutes (gray circle) and the cell viability was assessed 24 h later by neutral red assay. The percentage of viable cells was calculated in relation to control (untreated), considered as 100 %. The data are presented as mean ± S.E.M. of three independent experiments.
